# The Importance of Direct Progeny Measurements for Correct Estimation of Effective Dose Due to Radon and Thoron

**DOI:** 10.3389/fpubh.2020.00017

**Published:** 2020-02-11

**Authors:** Guillaume Samuel Bineng, Saïdou, Shinji Tokonami, Masahiro Hosoda, Yvette Flore Tchuente Siaka, Hamadou Issa, Takahito Suzuki, Hiromi Kudo, Oumarou Bouba

**Affiliations:** ^1^Nuclear Physics Laboratory, Faculty of Science, University of Yaounde I, Yaounde, Cameroon; ^2^Nuclear Technology Section, Institute of Geological and Mining Research, Yaounde, Cameroon; ^3^Department of Radiation Physics, Institute of Radiation Emergency and Medicine, Hirosaki University, Hirosaki, Japan; ^4^Department of Radiation Science, Hirosaki University Graduate School of Health Sciences, Hirosaki, Japan

**Keywords:** radon, thoron, progeny, equilibrium factor, effective dose

## Abstract

Radon (Rn), thoron (Tn), and thoron progeny (TnP) were measured in seven inhabited areas of the uranium and thorium bearing region of Lolodorf, located in southwestern Cameroon. Then the equilibrium factor (F_Tn_) between thoron and its progeny was determined in order to show the importance of direct progeny measurements for correct estimation of effective dose due to radon, thoron and their progenies. A total of 220 RADUET detectors were used to measure indoor radon and thoron and 130 TnP monitors for thoron progeny indoors. The arithmetic and geometric mean concentrations of Rn, Tn, and TnP were 103 and 89 Bq m^−3^, 173, and 118 Bq m^−3^, 10.7, and 7.4 Bq m^−3^, respectively. Total effective dose determined from radon, thoron, and their progenies was estimated at 4.2 ± 0.5 mSv y^−1^. Thoron equilibrium factor varied according to seasons, the type of dwelling, building materials and localities. Thoron (Tn and TnP) contribution to effective dose ranged between 3 and 80% with the average value of 53%. Total effective dose estimated from the world average equilibrium factor of 0.02 given by UNSCEAR was 2.7 ± 0.2 mSv y^−1^. The effective dose due to thoron varied greatly according to the different values taken by F_Tn_ and was different from that determined directly using TnP concentrations. Thus, effective dose due to thoron determined from the equilibrium factor is unreliable. Therefore, the risk of public exposure due to thoron (Tn and TnP) may therefore be higher than that of radon (Rn and RnP) in many parts of the world if F_Tn_ is no longer used in estimating total effective dose. This is not in contradiction with the UNSCEAR conclusions. It is therefore important to directly measure the radon and thoron progeny for a correct estimate of effective dose.

## Introduction

Radon is a noble gas. It is therefore supposed to have no affinity with other chemical elements. On the other hand, its solid disintegration products possess a great power of affinity with the material present in their environment. Among these dangerous descendants of radon, are polonium, bismuth, thallium, and lead. Through its descendants above, it is recognized as the second most responsible for lung cancer after smoking. Radon has three naturally occurring isotopes including radon (^222^Rn), thoron (^220^Rn) and action (^219^Rn) from ^238^U, ^232^Th, and ^235^U present in the Earth's crust, respectively, and a small amount from building materials (1–3).

Among the errors that are desirable to avoid nowadays in radioprotection, there is that of wanting to evaluate the risk of internal exposure of the public on the basis of the indirect determination of thoron effective dose from its equilibrium factor. Literature shows that there is no real correlation between the concentration of thoron gas and that of solid progeny in a dwelling (4). Thoron concentration in a dwelling depends on the distance from the source and the measurement result depends on the detector position in relation to the source. Near the wall and the ground, the concentration is high. Thus, the value of the concentration of thoron progeny determined from that of the gas thoron and the equilibrium factor is not reliable. This assertion also seems verifiable for radon and its progeny (5, 6). As a result, the risk of moving away from reality using the traditional approach above is so great that the total effective dose may be underestimated in some cases, or overstated in others. In practice, only a mere coincidence or pure chance can lead to the real result in certain circumstances. Before the advent of thoron progeny monitors in metrology, thoron dose was poorly known and its contribution in the total effective dose undervalued. Radon and thoron current knowledge, as well as experimental data collected on many sites where thoron progeny monitors have been deployed, challenge researchers on the true contribution of thoron to the total effective dose. Recent studies have shown that, the contribution of thoron to the internal exposure of the public is not always negligible compared to radon. In some places, this contribution may be greater than that of radon (4, 5, 7–11). The objective of the current study is to show the importance of direct progeny measurements for correct estimation of effective dose. For this, radon, thoron, and thoron progeny were directly measured using RADUET detectors and thoron progeny monitors to better estimate the total effective dose received by members of the public living in the uranium and thorium bearing region of Lolodorf, Cameroon. Likewise, thoron equilibrium factor was measured.

Total effective dose determined directly from radon, thoron, and the thoron progeny was compared with the doses estimated from the value 0.02 of the thoron equilibrium factor given by UNSCEAR (2), and the equilibrium factor measured in this work, respectively. The current study will contribute to confirm the UNSCEAR (12) conclusions about the important contribution of thoron to the effective dose in certain circumstances.

## Materials and Methods

### Study Areas

**Figure 1:**
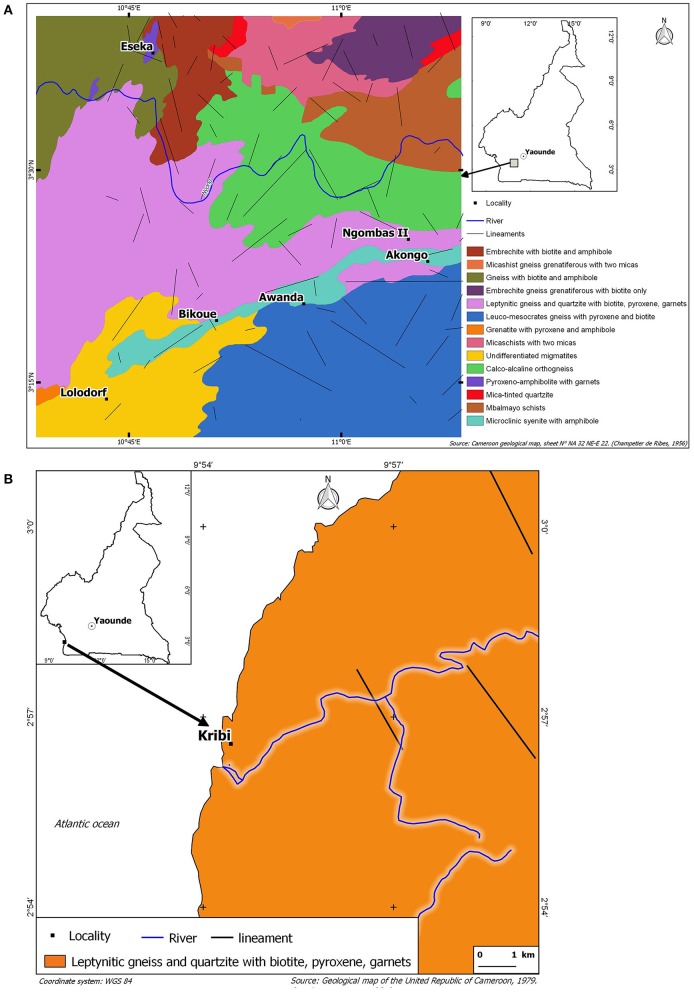
Geological map of the seven study areas located in the uranium and thorium bearing region of Lolodorf in West-southern Cameroon: the localities of Akongo, Awanda, Bikoue, Eseka, Lolodorf and Ngombas in **(A)**, and the locality of Kribi in **(B)**.

The uranium and thorium bearing region of Lolodorf as shown in Figures 1A,B is located in southwestern Cameroon, respectively, in the Ocean, the Nyong and Kelle, the Nyong and So'o Divisions. Eseka (E10°46′, N3°39′), Awanda (E10°59′, N3°22′), Ngombas (E11°06′, N3°25′), Akongo (E11°03′, N3°14′), Bikoue (E10°51′,N3°21′), Lolodorf (E10°44', N3°14'), and Kribi (E9°55', N2°57') are all approximately located between 70 to 340 km of Yaounde, the Capital city of Cameroon. It belongs to the Pan-African chain of Central Africa, more exactly in the Yaounde-East and Yaounde-West groups (13, 14). Literature shows that some rocks such as syenite, granite, granulites, ryolites, and plutonic can have high U and Th contents; radioactivity in diorites, basalts and gabbros is also significant (15, 16). In sedimentary rocks, the black shale, gypsum and anhydrides contain U and Th; the U content is high into the limestone. There is also radioactivity in sandstone, gravel, sand. Radioactivity may appear as an inclusion in the essential minerals, which are important constituents of rocks, or into accessory minerals (15–17). A detailed mineralogical study of Figure 1 (Geological maps of the seven study areas) shows that the soil and the bedrocks of the uranium and thorium bearing region of Lolodorf consists of the rocks and minerals mentioned above (13, 14, 16). The climate is equatorial; the West side is influenced by the proximity of the sea. Temperatures range from 25 to 26°C with two dry seasons between December-February and July-August, and two rainy seasons between September-November and March-June. The annual rainfall range is 1,500–2,000 mm, with a relative humidity of 70–80% recorded throughout the year (18). According to Central Bureau of the Census and Population Studies, the population is about 164,829 (19). The study area selected is of importance because it is known as having uranium and thorium anomalies at some specific places.

### Characteristics of the Studied Houses

Selection of dwellings in a locality was based on a double demographic and architectural criterion. Houses having a large number of occupants were prioritized. On the other hand, earth, mud, and bricks are the main building materials of the surveyed houses. In the villages, most of the walls are covered by soil and the floors consisted of ground. In the towns, most of houses were built using soil or cement bricks. The wall and floor of these houses were covered with a layer of concrete. In general, the primary target was residential areas that could be potential sources of radon and thoron. Measurements have been made preferably in rooms where residents spend a lot of time; some measurements were also carried out in the living room. Some houses had windows regularly closed or non-existent leading to a bad air circulation.

### Methods

Materials and methodology used in this study are well-described by Tokonami et al. (20). Each RADUET detector for integrated radon and thoron measurement was associated with a thoron progeny detector. All were hung at the same point on a wire placed 20 cm from the wall and 150 cm from the ground, far from windows, doors, and the source of heat and humidity that can influence the results. The first set of measurements was made through RADUET deployment for 2 months from March to May 2014 (in rainy season). A number of 90 RADUET detectors (Radosys Co. Ltd., Hungary) were deployed in 90 dwellings to measure simultaneously indoor radon and thoron only. The second set of measurements was carried out from January to March (dry season) and from June to August (rainy season) 2016. A number of 130 RADUET detectors and 130 thoron progeny monitors were simultaneously deployed in 130 dwellings to measure radon, thoron, and thoron progeny concentrations. In the current study, the period from June to August 2016 was considered a rainy season due to the region's heavy precipitation during the deployment of the measuring devices.

The CR-39 chips from RADUET depositions rate detector were chemically etched for 24 h in a 6 M NaOH solution at 60°C (4). Photographs of the formed alpha tracks were taken by digital camera using a microscope. Then, the number of alpha tracks on each photo was evaluated using IMAGE-J which is a public domain, a JAVA-based image processing program developed at the National Institutes of Health. Using alpha track densities of low and high air-exchange rate chambers, average radon and thoron activity concentrations were calculated as follows (21):

The average radon ($\bar{C_{Rn}}$) and thoron ($\bar{C_{Tn}}$) concentrations using alpha track densities of low and high air-exchange rate chambers were calculated as follows (21):

(1) $$\begin{array}{l} {\bar{C}}_{Rn}=\left(d_{L}-\bar{b}\right)\times \frac{f_{Tn2}}{t\;\times \;\left(f_{Rn1}\;\times {\;f}_{Tn2}-f_{Rn2}\;\times \;f_{Tn1}\right)}\\ \;-\left(d_{H}-\bar{b}\right)\times \frac{f_{Tn1}}{t\;\times \left(f_{Rn1}\times f_{Tn2}-f_{Rn2}\times f_{Tn1}\right)} \end{array}$$

(2) $$\begin{array}{l} \bar{C_{Tn}}=\left(d_{H}-\bar{b}\right)\times \frac{f_{Rn1}}{t\;\times \left(f_{Rn1}\times f_{Tn2}-f_{Rn2}\times f_{Tn1}\right)}\\ \;-\left(d_{L}-\bar{b}\right)\times \frac{f_{Rn2}}{t\;\times \left(f_{Rn1}\times f_{Tn2}-f_{Rn2}\times f_{Tn1}\right)} \end{array}$$

Where *d*_*L*_ and *d*_*H*_ were alpha track densities (track cm^−2^) for the low and high air-exchange, respectively. *f*_*Rn*1_and*f*_*Tn*1_, were the respective conversion factors from alpha track to ^222^Rn and ^220^Rn activity concentration in a low air-exchange rate chamber [(tracks cm^−2^h^−1^)/(Bq m^−3^)]. *f*_*Rn*2_ and *f*_*Tn*2_ were the respective conversion factors from alpha track densities to ^222^Rn and ^220^Rn in a high air-exchange rate chamber [(tracks cm^−2^h^−1^)/(Bq m^−3^)]. $\bar{b}$ was track density due to background (track cm^−2^) on the CR-39 detector and *t* was the sampling duration (h). The lower detection limits were 10 Bq m^−3^ for radon and 20 Bq m^−3^ for thoron.

Similarly, for calculating the equilibrium equivalent thoron concentration (EETC), the obtained track density was substituted in the following equation:

(3) $$\begin{array}{l} N_{TnP}=EETC\times F_{TnP}\times T+N_{B2\;} \end{array}$$

Where *N*_*TnP*_ was the background track density of CR-39 in the thoron progeny deposition detectors, *N*_*B*2_ was the background track density, *T* the exposure time and *F*_*TnP*_ the conversion factor for the thoron progeny deposition detectors. From results of a field survey (22) and the chemical etching conditions, value of *F*_*TnP*_ was 6.9 × 10^−2^ tracks *cm*^−2^ (Bq m^−3^ h)^−1^. The detection limit of EETC was <0.01 Bq m^−3^ for a measurement period of about 6 months.

The equilibrium factor (F) determines the level of radioactive equilibrium between radon, thoron, and their short-lived radioactive decay products, which is assumed to be 0.4 for radon and 0.02 for thoron (2). For this study, only the equilibrium factor for thoron was calculated from field data. The radon progeny concentration was calculated using the following formula:

(4) $$\begin{array}{l} EERC=C_{Rn}\times F_{Rn} \end{array}$$

Where *C*_*Rn*_ is the radon gas concentration in Bq m^−3^.

The equilibrium factor *F*_*Tn*_ of thoron can be determined as:

(5) $$\begin{array}{l} F_{Tn}=\frac{EETC}{C_{Tn}}. \end{array}$$

Where $C_{Tn}\left(Bq\;m^{-3}\right)$ is the thoron gas concentration.

#### Effective Dose Assessment Due to Indoor Radon and Thoron

Radon and thoron doses were determined according to two processes: the direct measurement illustrated by formula (7) using EETC directly measured from TnP (thoron progeny) monitors deployed on the site; then, the traditional or indirect measurement that uses the equilibrium factor between the gas and its progeny, as illustrated by formula (6).

Total effective doses of radon and its progeny (*D*_*Rn*_), thoron and its progeny (*D*_*Tn*_) has been calculated using the conversion factors for radon concentration (*C*_*Rn*_), thoron concentration (*C*_*Tn*_), EERC and EETC which are 0.17, 0.11, 9, and 40 nSvBq^−1^h^−1^m^3^, respectively, by the following equations (2):

(6) $$\begin{array}{l} D_{Rn}\left(\text{mSv }{\text{y}}^{-1}\right)\\ \;=\left(0.17+9\times F_{Rn}\right)\times C_{Rn}\times 8760\times 0.6{\times 10}^{-6} \end{array}$$

(7) $$\begin{array}{l} D_{Tn}\left(\text{mSv }{\text{y}}^{-1}\right)\\ \;=\left(\left(0.11\times C_{Tn}\right)+\left(40\times EETC\right)\right)\times 8760\times 0.6{\times 10}^{-6} \end{array}$$

Where 0.6 represents the indoor occupancy factor, 8,760 h (24 h × 365 days) is the time spent in a dwelling in 1 year. The occupancy factor usually used is 0.8. However, the current study has been made in Sub-Saharan Africa. Thus, temperatures are generally high; the minimum temperatures rarely reach 20°C in the shade. Most of the people spend the whole day working in farms, in the market, in the open air. Others who do not go to work spend more time outdoors, under the trees and verandas of dwellings because of the heat. Thus, if in temperate regions the public spends 80% of the time indoors, certainly because of the cold weather, in the current study, the time spent inside a house is estimated at 60%, which is an average of 14 h per day because of the heat, poverty, lack of air conditioner and lack of electricity.

The contributions from radon and thoron gas as given in Equations (6) and (7) above are not taken into account in the current study. The total effective doses due to radon (*D*_*Rn*_), and thoron (*D*_*Tn*_) were calculated using the formulas:

(8) $$\begin{array}{l} D_{Rn}=9\times F_{Rn}\times C_{Rn}\times 8760\times 0.6{\times 10}^{-6} \end{array}$$

(9) $$\begin{array}{l} D_{Tn}=40\times EETC\times 8760\times 0.6{\times 10}^{-\;6} \end{array}$$

Since the EERC was not measured directly on site in this work, the UNSCEAR (2) equilibrium factor F_Rn_ = 0.4 for radon was applied at all radon measurements points to take into account of the contribution of its progeny. For thoron contribution, only thoron progeny concentrations (EETC) were used instead of thoron gas concentrations for dose estimation because thoron concentration depends on many parameters among which the distance from wall/floor surface.

## Results and Discussion

### Radon, Thoron, and Thoron Progeny Concentrations

Radon, thoron and thoron progeny concentrations were determined for 174 over the 220 surveyed houses. The main results are shown in Tables 1–3. Table 1 summarizes these results.

**Table 1:** Summary of the results on radon, thoron, and thoron progeny survey: N is the number of surveyed houses.

**Radionuclide**	**AM ± SD** **(Bq m^**−3**^)**	**GM (GSD)** **(Bq m^**−3**^)**	**Median** **(Bq m^**−3**^)**	**Min–Max**
Radon (*N* = 173)	103 ± 2	89 (2)	91	28–976
Thoron (*N* = 130)	173 ± 13	118 (6)	141	23–724
EERC (*N* = 173)	41 ± 1	36 (2)	36.4	11–390
EETC (*N* = 95)	10.7 ± 0.9	7.4 (4.8)	7.7	0.4–37.6

*AM, arithmetic mean; GM, geometric mean; SD, standard deviation; GSD, geometric standard deviation; Min, minimum value; Max, maximum value*.

**Table 2:** Variation of indoor radon, thoron, and their associated progeny concentrations, thoron equilibrium factor in different types of house floor in seven inhabited areas of Lolodorf region, Cameroon: N is the number of houses surveyed.

	**The houses where the floors consisted of earth**					**The houses where the floors consisted of concrete**				
	**Rn con.** **(Bq m^**−3**^)**	**EERC** **(Bq m^**−3**^)**	**Tn con.** **(Bq m^**−3**^)**	**EETC** **(Bq m^**−3**^)**	**F_**Tn**_**	**Rn con.** **(Bq m^**−3**^)**	**EERC** **(Bq m^**−3**^)**	**Tn con.** **(Bq m^**−3**^)**	**EETC** **(Bq m^**−3**^)**	**F_**Tn**_**
N	93	93	64	23	23	90	90	66	72	62
AM	100	40	252	16	0.09	108	43	96	9	0.15
SD	11	4	18	2	0.01	4	1	12	1	0.02
GM	80	32	209	13	0.07	102	41	68	6	0.1
GSD	1	1	2	1	1.37	1	1	2	3	3.1
Med	69	28	227	16	0.07	115	46	56	6	0.1
Min	28	11	38	3	0.03	50	20	17	1	0.01
Max	976	390	724	38	0.21	197	79	420	38	0.85

**Table 3:** Seasonal variation of indoor radon, thoron, and their progeny concentrations along with thoron equilibrium factor: N is the number of houses surveyed.

		**N**	**AM ± SD**	**GM (GSD)**	**Median**	**Min–Max**
First set. Dry season 2014	Rn con. (Bq m^−3^)	93	86 ± 9	71 (1)	65	28–976
	EERC (Bq m^−3^)		34 ± 4	29 (2)	26	11–390
	Tn con. (Bq m^−3^)	59	184 ± 19	121 (1)	154	17–724
	EETC (Bq m^−3^)		Thoron progeny was not measured			
Second set. Dry season 2016	Rn con. (Bq m^−3^)	28	80 ± 3	78 (1)	79	52–121
	EERC (Bq m^−3^)		32 ± 1	31 (1)	32	21–48
	Tn con. (Bq m^−3^)	26	57 ± 7	49 (1)	49	17–157
	EETC (Bq m^−3^)	29	9 ± 1	7 (2)	7	2–30
	F_Tn_		0.25 ± 0.05	0.15 (2.8)	0.17	0.02–0.85
Second set. Rainy season 2016	Rn con. (Bq m^−3^)	52	131 ± 3	130 (1)	131	90–197
	EERC (Bq m^−3^)		52 ± 1	52 (1)	52	36–79
	Tn con. (Bq m^−3^)	45	149 ± 18	105 (2)	109	18–451
	EETC (Bq m^−3^)	56	11 ± 1	7 (1)	7	1–38
	F_Tn_		0.09 ± 0.01	0.08 (1.24)	0.08	0.01–0.21

The direct values were those obtained experimentally on the site using RADUET detectors and thoron progeny monitors, while the estimated values were those using the factors F_Rn_ = 0.4 for radon, F_Tn_ obtained on the different sites and F_Tn_ = 0.02 given by UNSCEAR (2).

**Figure 2:**
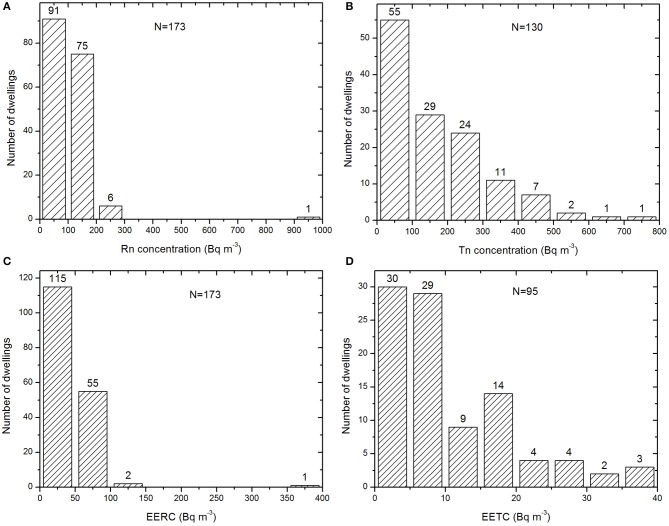
Frequency distribution of radon **(A)**, thoron **(B)**, RnP **(C)**, and TnP **(D)** in some dwellings.

Figure 2 displays the frequency distribution of radon, thoron, RnP, and TnP in some dwellings. Radon, thoron, and their progeny concentrations determined in the field were distributed asymmetrically. The reason is that the low values were much more numerous than the large ones. As a result, the high extreme values strongly influence the arithmetic mean of such a distribution. This distribution of measurements was expected in this study because it concerns the results of a one-off sampling (short duration).

In Table 1, the radon concentration varied from 28 to 976 Bq m^−3^ with an arithmetic mean of 103 ± 2 Bq m^−3^ and geometric mean (and standard geometric deviation) of 89 (2) Bq m^−3^. These values were two both lower than 300 Bq m^−3^, the reference value recommended by the International Atomic Energy Agency (23) and the International Commission on Radiological Protection (3). Indeed, only one dwelling over 173 monitored had a radon concentration >300 Bq m^−3^. This dwelling in the locality of Bikoue is located at a place where anomalies of uranium and thorium have been detected (24). It is in the same house that the highest concentration of thoron was measured. This could also be the case with associated descendants of thoron, if the detector had been read. Compared to the world average value of 45 Bq m^−3^, the indoor radon concentrations (arithmetic and geometric means) obtained in the current study were 4 and 2 times higher, respectively (2). In addition, 47% of houses had radon concentration higher than 100 Bq m^−3^ which is a reference value recommended by the World Health Organization (1). Similarly, 8% of dwellings had indoor radon higher than the action level of 148 Bq m^−3^ recommended by the USEPA (25) and 4% of dwellings had indoor radon higher than the action level of 200 Bq m^−3^ prescribed in many European Union countries (26). For thoron, the world average value is 10 Bq m^−3^ (2). The thoron concentration exceeded 100 Bq m^−3^ in 43% of houses, while 13% had a concentration higher than 300 Bq m^−3^. So, no dependence is evidenced between indoor radon and thoron concentrations in the dwellings. It should be noted that the reference level of 100 Bq m^−3^ is only valid for radon. No reference value is yet defined for thoron. Nevertheless, more than half of the houses (54%) have thoron concentrations exceeding 100 Bq m^−3^, 17 times higher than the average world value given above. No house had a concentration lower than the worldwide average value.

EETC were determined according to two processes: the first one is essentially based on the direct results coming from the TnP dosimeters deployed on the site, and the second one, on the data resulting from thoron measurements. The latter case is an indirect method.

The results of the direct measurements of the EETC ranged from 0.4 to 37.6 Bq m^−3^ with an arithmetic mean of 10.7 Bq m^−3^. This mean value was about 22 times higher than 0.5 Bq m^−3^, which is the world average value (2). Only 1% of houses had concentration lower than the world average value given above. EETC in 68% of dwellings exceeded 5 Bq m^−3^, 10 times the world average value. Geometric mean (7.4 Bq m^−3^), arithmetic mean and the maximum value were high. However, the maximum value was not completely isolated from the other values of the distribution. The maximum EETC value was measured in an Awanda dwelling where the thoron and radon concentrations were 380 Bq m^−3^ and 134 Bq m^−3^, respectively. Fifty one percent of dwellings were higher than 7.7 Bq m^−3^; 5% of dwellings had EETC >30 Bq m^−3^, and 3% have EETC above 37 Bq m^−3^.

Average value of EERC (41 Bq m^−3^) was approximately 3 times higher than the world average value of 15 Bq m^−3^ (2). ninety eight percentage of 173 surveyed dwellings had EERC higher than the world average value. In addition, 47% of houses had EERC exceeding 100 Bqm^−3^, 4% above 200 Bq m^−3^ and 1% above 300 Bq m^−3^.

Most of values lower than 100 Bq m^−3^ were located in the dwellings of Awanda, Akongo, Kribi, Lolodorf and Ngombas for radon, and Bikoue, Akongo, Ngombas, Lolodorf, and Awanda for thoron. Moreover, the highest concentrations were found at Bikoue. Concerning EETC, high concentrations were found in all sites.

**Figure 3:**
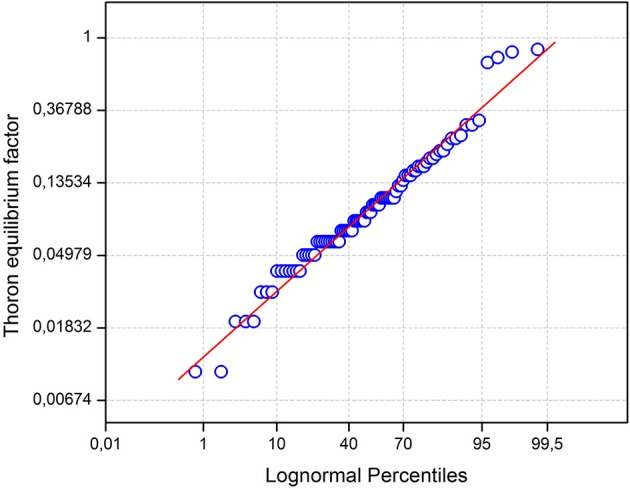
Probability plots of the equilibrium factor (*N* = 85).

**Figure 4:**
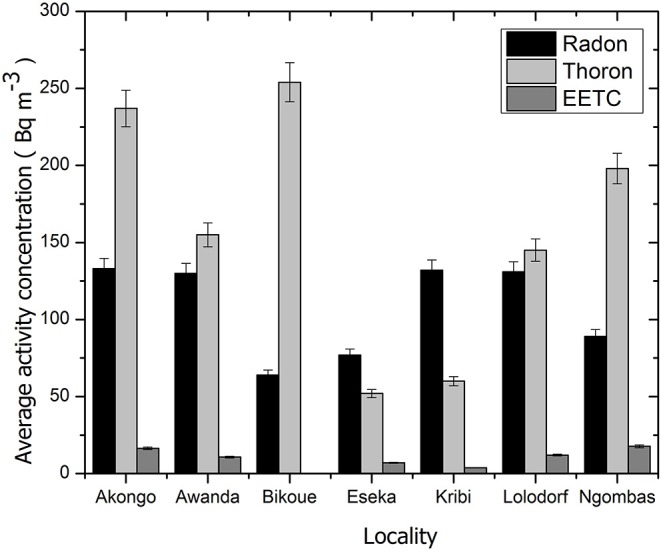
Comparison of average activity concentrations of radon, thoron and EETC for all localities.

Figure 3 shows the probability plots of the equilibrium factor. Correlation between thoron and its progeny is very poor of the study area. Figure 4 displays the comparison of geometric average activity concentrations of radon, thoron, and EETC for all inhabited areas. There was more thoron than radon in houses except in Eseka and Kribi. In effect, the sampling results in this study are distributed according to a log-normal distribution whose statistical parameters are GM and GSD. However, the GSDs show that the data are quite dispersed. This could be explained by the fact that: the measurement sites were very far apart from each other. The types of building materials, the geological specificities of the building construction place, the architecture and the lifestyles of the populations varied from one site to another. Some houses had windows regularly closed or non-existent. In addition, it should be noted that some measurements have been made in the houses built in areas where uranium and thorium anomalies have been discovered. This is the case of the locality of Bikoue where a dwelling revealed the highest concentrations (Rn: 976 Bq m^−3^; Tn: 724 Bq m^−3^, TnP: 10.7 Bq m^−3^) of the current study. The *in-situ* measurements in the courtyard of the above house revealed very high concentrations of ^238^U and ^232^Th (18 Finally, the RADUET detectors were not deployed during the same time (year/dry season/rainy season). But processing the data by season, type of dwelling or locality, this GSD could be less important for the whole study.

As all experimental methods, the methods used in the current study measuring radon, thoron, and thoron progeny in dwellings also have their limitations. The values of the uncertainties observed in some results effectively justify these limits. RADUET detectors and thoron progeny monitors are solid state nuclear track detectors (SSNTD). That, are samples of a solid material exposed to nuclear radiation, etched and examined under a microscope. Their aging effect, the direct exposure of a sensor to solar radiation, heat and humidity are the real sources of uncertainty during the measurements (21). In addition, it is known that indoor EETC and thoron concentration can fluctuate temporarily and spatially extensively. Thus, their uncertainty will propagate into the calculation of the F_Tn_.

In practice, the detectors are deployed in dwellings for a relatively long time, away from the control of the practitioner. Only the common sense and cooperative spirit of the residents of the surveyed house can lead to reliable results of the measurements. If the detectors are manipulated by a dwelling resident in the absence of the practitioner (scraped, exposed to sources of heat or humidity), then the results of the measurements will undoubtedly be influenced. Beyond the deviations linked to handling as well as to electronic and chemical processing in the laboratory, the uncertainties observed on the different results (thoron equilibrium factor, the radon, thoron and thoron progeny concentrations) of this study can also be justified by the location of different detectors as well as the specificities of the surveyed houses.

In the current study, the maximum radon and thoron concentrations are very high and almost isolated from other values in the distribution. Thus, other measurements should be made at these particular points in order to better validate the estimate of the risk incurred by the resident public. Statistically, these values greatly increase the uncertainty about the mean concentration for the entire study area. For the EETC, this is a series of high values, because 37% of dwellings have an EETC >10.7 Bq m^−3^ and 51% are higher than 7.7 Bq m^−3^. It is therefore better to take into account all of them in the calculations. The characteristics of the dwellings selected in this study also justify their high values. In addition, previous study carried out in the same study areas of this study revealed the presence of the anomaly of ^232^Th and ^238^U at some specific points in the region (24, 27, 28). However, the literature (29) has revealed a strong link between EETC on the one hand, and type of architecture, geology, climate and people's lifestyle on the other. In this study, the average concentrations of thoron and its progeny in the whole region are high (118 and 7.4 Bq m^−3^, respectively). These geometric means are 12 and 15 times higher than their corresponding world average values, respectively, and also confirm the presence of ^232^Th in the area at relatively large proportions. It is clear from the current study that, there is a strong correlation between public exposure to thoron progeny and high concentrations of ^232^Th in soil/building materials in a dwelling. As a result, the public living in these dwellings is exposed to associated radiation and radiation protection measures should therefore be considered to reduce this exposure. These results are therefore in perfect agreement with the conclusions of UNSCEAR (12) and ICRP (29) on the possibility of radiological protection problems due to thoron exposures in some particular cases of houses. In practice, each member of the public is individually exposed to natural radioactivity. This exposure depends on his lifestyle, the living in environment and the work place. This means that the average concentration does not give information on the dose received by each individual permanently living in the region current study area. This concentration is simply a tool that informs public opinion about the evolution of population exposures over the years. The values of these mean concentrations represent an estimate that is subject to many uncertainties and fluctuations. To individualize them, the specificities and environmental characteristics of the person should be taken into account.

It should be noted that, the coefficient of variation (CV) is defined as the ratio of the standard deviation by the mean of a series of measurements. It provides information on the dispersion of values around the mean. The coefficients of variation are usually linked to measurement instruments and analytical methods. The lower the CV value, the more accurate the estimate. In the current study, the CV of the concentration values was 1.9, 2.4, and 8.4% for Rn, Tn and TnP, respectively. All these values are <10%; therefore, their estimate is good (30). Statistically, these results are therefore reliable.

Table 2 displays the variation of indoor radon, thoron and their associated progeny concentrations, thoron equilibrium factor in different types of house floor. In Table 3 one can notice the seasonal variation of indoor radon, thoron and their progeny concentrations along with thoron equilibrium factor. In the above tables, there was not a big difference between indoor radon levels in both types of homes. On the other hand, dwellings with earthen floor had more thoron and thoron progeny than those with concrete floor. On the rainy season, there was more radon and thoron in the dwellings than the dry season. As for thoron progeny, they varied slightly from one season to another. All this could be justified by the fact that when it rains, weather is in general cold and the dwellings remain closed for a long time. As a result, radon, thoron, and their associated progeny accumulate, sometimes reaching high concentrations.

### Radiation Dose Due to Inhalation of Radon and Thoron

#### Effective Dose Using EETC: Direct Measurements

For the entire study area, radon and thoron gases contributions were, respectively, evaluated as 2% (0.09 mSv y^−1^) and 2.3% (0.1mSv y^−1^). For radon and thoron progenies, the contributions are 45% (1.96mSv y^−1^) and 51% (2.24 mSv y^−1^), respectively. Their contributions appearing too low, the doses of radon and thoron gases are negligible. In addition, once inhaled, these radionuclides are almost completely exhaled. On the other hand, their daughters, solid and radioactive particles, will be deposited on the bronchi and in the lungs. They will thus be able to cause an irritation of the cells of bronchial and pulmonary tissues which can induce a cancer. Thus, health risk of radon and thoron is not related to the gas itself, but to their daughter products (2). Therefore, taking into account of their different contributions in estimating the totaleffectivedose could lead to errors in the expected result. The average totaleffective dose of the entire study area was estimated at 4.2 mSv y^−1^ ranging between 0.53 and 18.47 mSv y^−1^. This total estimated dose due to indoor radon and thoron is approximately 3.5 times higher than the corresponding world average value.

**Figure 5:**
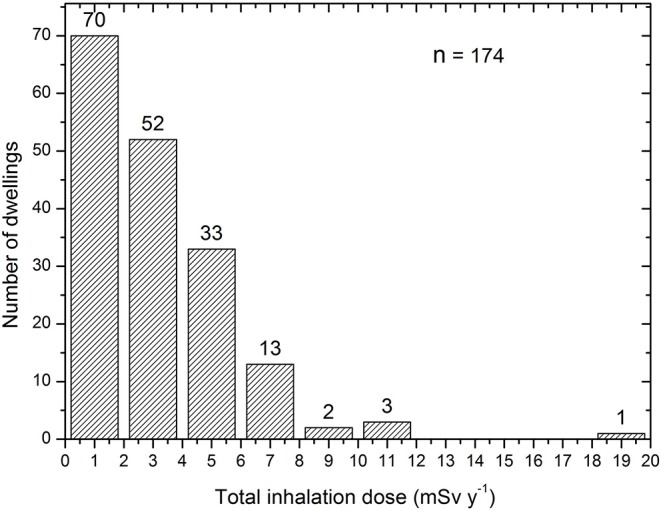
Distribution of total effective doses due to radon, thoron and TnP.

Figure 5 shows the distribution of the total effective doses due to radon, thoron and TnP while, the total effective dose due to radon and thoron was displayed in Table 4. This distribution represents the data from the seven studied areas. Though, 73% of surveyed houses exceed the worldwide average value of 1.26 mSv y^−1^ (2).

**Table 4:** Statistical parameters related to effective doses due to indoor radon and thoron of the whole of the seven study areas of the uranium and thorium bearing region of Lolodorf, Cameroun.

**Radionuclide**	**Mean** **(mSv y^**−1**^)**	**Median** **(mSv y^**−1**^)**	**Ranges** **(mSv y^**−1**^)**
Rn	0.09 ± 0.01	0.09	0.03–0.87
RnP	1.96 ± 0.12	1.81	0.53–18.47
Tn	0.1 ± 0.1	0.08	0.01–0.42
TnP	2.24 ± 0.19	1.62	0.08–7.91
Total effective dose	4.2 ± 0.3	4.5	0.56–18.47

Tables 5, 6 show the variation of effective dose according to the different types of house floor and the season, respectively. The first set of the measurements covered the localities of Eseka, Ngombas and Lolodorf (dry season, 2014). The second set covered only Eseka (dry season, 2016) and the localities of Akongo, Awanda, Kribi, and Ngombas (small rainy season, 2016). The mean indoor thoron concentrations for the three monitoring periods were 245, 32, and 149 Bq m^−3^, respectively. As shown in Table 6, when the value 0.02 of the equilibrium factor given by UNSCEAR (2) was used in the calculation, the associated thoron progeny effective doses were estimated at 1.03, 0.24, and 0.63 mSv y^−1^, respectively. These results probably would not have been the same if surveillance covered all seven localities during each period. As illustrated in Figure 5, the effective doses due to radon and thoron in the various inhabited localities are independent each another. The members of the public living in the uranium and thorium bearing region of Lolodorf are more exposed in dwellings with earthen floors. In the same way, they are more exposed in rainy season than in dry season. This is due to the accumulated concentrations of radon, thoron and their associated progeny in dwellings.

**Table 5:** Variation of annual effective dose rate due to indoor radon, thoron, and their associated progeny in different types of house floor: D_Rn_, D_RnP_, D_Tn_,D_TnP_, and D'_TnP_ are the effective doses due to radon, thoron, radon progeny, and thoron progeny respectively.

	**Houses where the floor consisted of earth**			**Houses where the floor consisted of concrete**		
	**AM ± SD** **(mSv y^**−1**^)**	**GM (GSD)** **(mSv y^**−1**^)**	**Ranges**	**AM ± SD** **(mSv y^**−1**^)**	**GM (GSD)** **(mSv y^**−1**^)**	**Ranges**
D_Rn_	0.09 ± 0.01	0.07 (1.32)	0.03–0.87	0.1 ± 0.1	0.09 (1.15)	0.04–0.18
D_RnP_	1.89 ± 0.21	1.52 (1.31)	0.53–8.47	2.03 ± 0.07	1.93 (1.15)	0.93–3.73
D_Tn_	0.15 ± 0.01	0.12 (1.53)	0.02–0.42	0.06 ± 0.01	0.04 (1.74)	0.01–0.24
D_TnP_	3.35 ± 0.39	2.82 (1.46)	0.53–7.91	1.89 ± 0.21	1.28 (1.74)	0.08–7.88
D'_TnP_	1.06 ± 0.08	0.88 (1.53)	0.16–3.04	0.4 ± 0.1	0.28 (1.64)	0.07–1.77

*D'_Tn_ is the effective dose due to thoron using F_Tn_ = 0.02*.

**Table 6:** Seasonal variation of annual effective dose rate due to indoor radon, thoron, and their associated progeny.

**Season**	**Effective dose** **(mSv y^**−1**^)**	**AM ± SD**	**GM (GSD)**	**Median**	**Min–Max**
First set:dry season 2014	D_Rn_	0.08 ± 0.01	0.06 (1.17)	0.06	0.03–0.87
	D_RnP_	1.63 ± 0.18	1.35 (1.17)	1.24	0.53–18.47
	D_Tn_	0.11 ± 0.01	0.07 (1.92)	0.09	0.01–0.42
	D_TnP_	Thoron progeny were not measured			
	D'_Tn_	0.77 ± 0.69	0.51 (2.15)	0.65	0.07–3.04
Second set:dry season 2016	D_Rn_	0.07 ± 0.01	0.07 (1.04)	0.07	0.05–0.11
	D_RnP_	1.51 ± 0.06	1.47 (1.04)	1.49	0.98–2.29
	D_Tn_	0.03 ± 0.01	0.03 (1.27)	0.03	0.01–0.09
	D_TnP_	1.88 ± 0.26	1.48 (1.54)	1.37	0.38–6.22
	D'_Tn_	1.38 ± 0.16	1.19 (1.32)	1.18	0.41–3.82
Second set: rainy season 2016	D_Rn_	0.12 ± 0.01	0.12 (1.03)	0.12	0.08–0.18
	D_RnP_	2.48 ± 0.05	2.46 (1.02)	2.48	1.7–3.7
	D_Tn_	0.09 ± 0.01	0.06 (2.72)	0.06	0.01–0.26
	D_TnP_	2.26 ± 0.31	1.39 (4.67)	1.41	0.08–7.91
	D'_Tn_	0.63 ± 0.07	0.44 (2.71)	0.46	0.08–1.9

*D_Rn_, D_RnP_, D_Tn_, D_TnP_, and D'_TnP_ are the effective doses due to radon, thoron, radon progeny, and thoron progeny respectively. D'_Tn_ is the effective dose due to thoron using F_Tn_ = 0.02*.

According to International Commission on Radiological Protection (3), effective dose due to thoron (Tn and TnP) is generally negligible compared to that from radon (Rn and RnP) in most areas of the world. But in the current study, it is the opposite, except Kribi where radon contribution is practically twice that of thoron. Otherwise, thoron (Tn and TnP) contribution to the total dose ranged between 3 and 80% with the average value of 53%. Therefore, thoron and its progeny should be taken into account when assessing radiation doses and health risks.

#### Effective Dose Using Different Thoron Equilibrium Factors: Indirect Measurement

As displayed in Tables 2, 3, thoron equilibrium factor can vary considerably from one site to another. It also varies according to seasons, the types of dwelling and building materials. Results showed that the value of 0.02 recommended by the United Nations Scientific Committee for the Study of the Effects of Atomic Radiation was very low (2).

**Figure 6:**
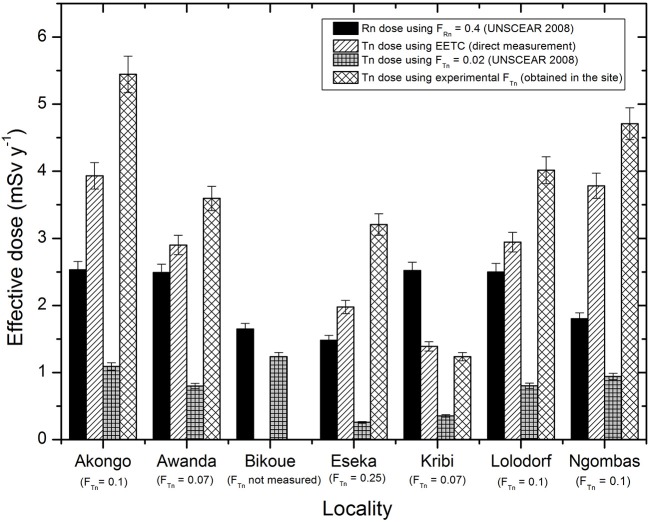
Comparison of inhalation dose of radon and thoron for all localities.

Using the equilibrium factor F_Tn_ = 0.02 given by UNSCEAR, effective dose due to thoron and its progeny would range from 0.07 ± 0.02 to 3.04 ± 0.75 mSv y^−1^ with an average value of 0.73 ± 0.05 mSv y^−1^; this average value is 3 times lower than that of 2.24 mSv y^−1^ obtained directly from EETC. Thus, the total effective dose would be 2.7 ± 0.2 mSv y^−1^; 1.6 time smaller than the direct dose above, estimated in this work. The specific case of each locality is presented in Figure 6 (comparison of effective dose of radon and thoron for all localities). Here, the dose of radon and those of thoron were compared in each inhabited area. In addition, this figure also compares thoron doses directly determined from EETCs and those estimated using the various equilibrium factors. Each inhabited locality is associated with its experimental average equilibrium factor between the thoron and its progeny. The total effective dose in a locality is the sum of the radon dose and the thoron dose specific to the type of measurement used (EETC or F_Tn_). This influence of the thoron equilibrium factor in the calculation of the effective dose can also be observed in Figure 6 as well as in Tables 5, 6. When the 0.02 equilibrium factor of UNSCEAR (2) is used, the inhalation dose of thoron decreases. On the other hand, when thoron equilibrium factor value specific to a locality, the variation of the seasons or the type of construction materials is used, the inhalation dose increases considerably, hence the importance of direct progeny measurements for correct estimation of effective dose.

The similar studies were conducted by Serge Didier et al. (8) and Ndjana Nkoulou et al. (9) in Douala and Betare-Oya gold mining areas, which are, respectively, two localities of Cameroon. The contribution of thoron and its progeny to the total effective dose was found to vary from 7 to 60% with an average value of 26% in Douala, and 7–70 with an average of 30 in Betare-Oya gold mining areas. In some dwellings in the High Levels Natural Radiation areas of Kerala, India, radon and thoron doses using radon and thoron progeny concentrations directly measured at the study sites were determined (5). The average equilibrium factor in dwellings was 0.51 ± 0.16 and 0.07 ± 0.04, respectively, for radon and thoron. The above values vary and the equilibrium factor of thoron was 3.5 times higher than that proposed by UNSCEAR. The dose ratio of thoron progeny to radon progeny is equal to 0.6. This means that the dose of thoron contributes significantly to the total inhaled dose. This certainly should not have been the case if equilibrium factors were used in the measurements of these doses. Similar work has been done by Omori et al. (11) in some localities of Kerala. The dose of radon progeny was measured using the equilibrium factor of 0.4; it ranged from 0.02 to 1.07 mSv y^−1^ with an arithmetic mean of 0.14 mSv y^−1^. The thoron progeny dose calculated directly from the thoron progeny deposition detectors deployed at the sites ranged between 0.10 and 2.24 mSv y^−1^ with an arithmetic mean of 0.55 mSv y^−1^; 4 times higher than that due to the descendants of radon. Certainly, the radon dose in this study is underestimated because the study area is the same and the equilibrium factor for the area is estimated at 0.51 by Mayya et al. (5). In addition, this dose could have been higher if the measurement was made directly from the detectors using the radon progeny deposition detectors. Recent study made by Kaur et al. (31) in the dwellings of Sub-mountainous region of Jamma and Kashmir in India revealed that the equilibrium factor of radon, thoron and their associate progenies ranged from 0.01 to 0.03 with the mean value of 0.02, and from 0.3 to 1 with a mean value of 0.6, respectively. Total effective dose due to radon, thoron, and their associate progenies, using EERC and EETC directly measured from TnP and RnP monitors deployed on the site was 1.2 mSv y^−1^. The thoron contribution in the total effective dose was 25%; which is not negligible from the exposure point of view.

It is well-known that the effective dose of thoron is generally negligible compared to that of radon in most parts of the world (32). Would this assertion not be related to the method used in the estimation of doses? If the value of 0.02 set by UNSCEAR is applied in the dose determination, then it is highly likely that the above assumption is still true. But if it is the equilibrium factor or the concentrations of thoron progeny directly measured at the study site that are used, then it becomes very difficult to prove the UNSCEAR statement above. This can be explained by the fact that thoron is a thorium progeny, which is more abundant on land than uranium; in addition, the dose conversion factor of thoron progeny (40 mSv Bq^−1^h^−1^m^3^) is 4 times higher than that of radon progeny (9 mSv Bq^−1^h^−1^m^3^). Thus, to estimate the dose inhaled by a public, the most effective and reliable method is probably the one that uses the radon progeny and thoron progeny concentrations measured directly on the study site using the detectors deployed for this purpose; this approach does not undervalue the dose, nor does it overvalue it.

From the foregoing, it is found that the use of the 0.02 value of the factor given by UNSCEAR (2) significantly underestimates the total effective dose by the public of the uranium and thorium bearing region of Lolodorf. Likewise, the use of any mean value of the equilibrium factor obtained directly on the site greatly overestimates the total effective dose. Consequently, the deviation associated with the estimation of the total inhaled dose in general and that due to thoron in particular is very great when an equilibrium factor is used in the calculations. So, the risk of public exposure to thoron, traditionally known, may be higher than that of radon in many parts of the world if all the above mentioned factors are taken into account in estimating the total effective dose.

## Conclusion

Total effective dose determined directly from thoron and its progeny was compared with the doses estimated using the equilibrium factor (F_Tn_) given by UNSCEAR, the F_Tn_ of houses having earthen and concrete floors, the F_Tn_ of the seasonal variations and the F_Tn_ measured in the seven study areas of the uranium and thorium bearing region of Lolodorf, respectively. Thoron effective dose, traditionally determined varied according to the equilibrium factor used, and remained different from that of the direct measurement which uses EETC. So, the internal radiation dose resulting from the direct measurement seems more realistic. It can better inform about the thoron contribution to the total effective dose due to radon and thoron. The risk of public exposure due to thoron may therefore be higher than that due to radon in many parts of the world if F_Tn_ is no longer used in estimating total effective dose. This study confirms the conclusions of UNSCEAR and ICRP on the important contribution of thoron and its associated progeny in the effective dose under some conditions.

## Data Availability Statement

The datasets generated for this study are available on request to the corresponding author.

## Author Contributions

Conception was made by S, TS, and OB. Data acquisition and analysis were made by GB, S, TS, MH, YT, ST, HK, and OB. Data interpretation was made by GB, MH, HI, ST, and HK. The article was written by GB and review by S, TS, MH, YT, HI, ST, and HK.

### Conflict of Interest

The authors declare that the research was conducted in the absence of any commercial or financial relationships that could be construed as a potential conflict of interest.
